# Modeling preferential attraction to infected hosts in vector-borne diseases

**DOI:** 10.3389/fpubh.2023.1276029

**Published:** 2023-11-22

**Authors:** Ishwor Thapa, Dario Ghersi

**Affiliations:** School of Interdisciplinary Informatics, University of Nebraska at Omaha, Omaha, NE, United States

**Keywords:** agent-based modeling, preferential attraction, vector borne disease, computational modeling, viral propagation model

## Abstract

Vector-borne infectious diseases cause more than 700,000 deaths a year and represent an increasing threat to public health worldwide. Strategies to mitigate the spread of vector-borne diseases can benefit from a thorough understanding of all mechanisms that contribute to viral propagation in human. A recent study showed that *Aedes* mosquitoes (the vectors for dengue and Zika virus, among others) are preferentially attracted to infected hosts. In order to determine the impact of this factor on viral spread, we built a dedicated agent-based model and parameterized it on dengue fever. We then performed a systematic study of how mosquitoes' preferential attraction for infected hosts affects viral load and persistence of the infection. Our results indicate that even small values of preferential attraction have a dramatic effect on the number of infected individuals and the persistence of the infection in the population. Taken together, our results suggests that interventions aimed at decreasing the preferential attraction of vectors for infected hosts can reduce viral transmission and thus can have public health implications.

## 1 Introduction

Vector borne diseases like dengue, malaria, and yellow fever are infections transmitted by the bite of blood-feeding arthropods such as mosquitoes and ticks. In 2019, 56,879 million cases of dengue were reported, with 36,055 deaths attributable to dengue globally (1). With the increasing global burden of dengue and other vector-borne diseases, it is critical to determine all the factors that influence the overall transmission of the virus. In this study, we developed a computational model to understand how the viral transmission can be influenced by vector attraction for infected hosts.

A recent study by Zhang et al. (2) showed that dengue and Zika infected mice were more attractive to the *Aedes* mosquitoes than uninfected mice. The study showed that dengue and Zika infections in mice lead to increased release of acetophenone, a mosquito attractant, due to altered skin microbiota (2). Interestingly, preference toward infected humans has also been observed in malaria, where mosquitoes are more attracted to humans in transmissible stage than to uninfected and infected individuals in non-transmissible stages (3). *Anopheles* mosquitoes are known malaria vectors and have been shown to respond to skin volatile compounds emitted by skin-associated bacteria in the hosts (4). In addition to skin microbiome, body odor and underlying health conditions can influence the mosquitoes' attraction to humans (5). Administering vitamin A to the host was able to reduce acetophenone release due to decreased abundance of *Bacillus* spp. in the skin microbiome, leading to decreased mosquito attraction (2). However, composition of the human skin microbiota is influenced by many factors including skin site, sex, age, and use of cosmetics (6). More studies are needed to study the efficacy of vitamin A in reducing the mosquito attraction in dengue-infected individuals.

While the study by Zhang et al. (2) focused on understanding the biochemical mechanisms that make infected hosts more attractive to mosquitoes, the impact of such mechanisms on virus transmission has not yet been fully elucidated. More specifically, how viral load and persistence of viral infection in the population can be impacted by changes in mosquitoes' attraction to infected hosts is still poorly understood. Knowing whether preferential attraction to infected hosts has a major effect on viral propagation in a population can have important implications for public health initiatives aimed at reducing the burden of vector borne diseases.

In order to study the effect on viral propagation when vectors are preferentially attracted to infected hosts, we built a simple SIR (7) agent-based model (ABM) with mosquitoes and human hosts as agents, and introduced a *bias* parameter that controls preferential attraction of mosquitoes to infected hosts. We designed computational experiments to systematically investigate the effect of the *bias* and other key parameters in the model on viral propagation and total viral load.

Agent-based models have been widely applied in biomedical sciences, with areas of focus ranging from immune system responses to viral infections (8, 9), tumor development and response to different therapies including immunotherapy (10, 11), and viral transmission during pandemics (12). ABMs are comprised of individual agents, with well-defined behavior and rules of interactions, and are an orthogonal approach to equation-based mathematical modeling (13). In both types of approaches, choosing a correct set of parameters is key to successfully model the phenomenon of interest (14), (15), (16), (17). In this study, we specifically parameterized the model using data from the literature on dengue virus. However, the model can be easily adapted to simulate other vector borne diseases like malaria or Zika. Using this dengue infection model, we show that preferential attraction to infected hosts plays a key role in sustained infections. Without any preference for infected hosts (i.e., with the *bias* parameter set to 0), the viral load is minimal and the infection dies out quickly in the population. Taken together, these results suggest that interventions designed to interfere with mosquito preference for infected hosts could be effective for controlling vector-borne diseases.

## 2 Methods

In this section, we describe the agent-based model that we developed using the NetLogo language (18), and discuss the computational experiments and analyses performed with the model.

### 2.1 NetLogo model

1. *Agents and their properties:* The two agents in the model are mosquitoes and humans. Human agents have three states, viz. uninfected, infected, and recovered. Similarly, mosquitoes have three states, viz. uninfected, incubation, and carrier.
2. *Agents' behavior:* At every tick (time step), mosquitoes, and humans move. Human agents can move in any direction randomly, while the direction of mosquitoes depend on the *attractiveness* and *bias* parameter values. *Attractiveness* can be defined as the probability that a mosquito will move toward the closest human. *Bias* can be defined as the probability that a mosquito will move toward the closest infected human, given that the mosquito is attracted to human. In short, the *attractiveness* represents mosquitoes' attraction to human agents, whereas *bias* represents preferential attraction to infected individuals.
3. *Environment:* World of 33 by 33 patches with toroidal topology.
4. *Interactions:*
  Mosquitoes: When an uninfected mosquito is in the same location as an infected host, the mosquito is inoculated with the virus and its state changes to incubation stage. Average incubation time is set to 7 days based on published data on dengue transmission (19). At the end of the incubation time, the state of the mosquito will change to carrier state, and the mosquito can now infect an uninfected human. The mosquito remains in carrier state for its lifetime, which is set to 21 days (see Table 1). After its lifetime (determined by average carrier time), the mosquito reverts to the uninfected state, simulating the replacement of a mosquito with a newly born one.
  Humans: Once a human agent is infected by the bite of a carrier mosquito, its state changes to infected stage and remains sick for a period of time. Average sick time is set to 5 days (see Table 1). After the sick time is over, the individual can no longer transmit the virus to another mosquito and cannot get re-infected for a period of time. Based on multiple studies, the average time before the individual can be re-infected is set to 30 days (see Table 1).
5. *Model assumptions:* The model assumes constant human and mosquito populations throughout the simulation, i.e., the death rate equals the birth rate.
6. *Output variables:* At every time point, viral load is measured as the sum of the counts of infected humans and mosquitoes in incubation and in carrier stage.
7. *Stopping criterion:* If all humans and mosquitoes are clear of infection, the simulation stops. In cases when the infection continue to persist in the population, the simulation has a hard stop at 2,000 ticks.

**Table 1:** Typical model parameter values with sources of information on dengue specific parameters.

**Parameter**	**Value (s)**	**Source (s)**
Number of individuals	100	–
Number of mosquitoes	100*	–
Percentage infected at start	10*	–
Attractiveness	0.1*	–
Bias	0.1*	–
Average sick time	5 (3–7) days	Simmons et al. (20)
Average carrier time	21 (10–35) days	Goindin et al. (21)
Average incubation time	7 (3–10) days	Kularatne (19)
Average recovery time	30 days	Nishiura (22) (1–2 weeks) and Snow et al. (23) (8 weeks)

The star (*) indicates parameters that are directly explored in the study.

#### 2.1.1 Model parameters

There are three types of parameters in the NetLogo interface (see Supplementary Figure 1): (1) Initial population sizes of mosquito and human and percentage of infected mosquito; (2) dengue infection specific parameters such as average sick time, carrier time, incubation time, and recovery time; and (3) human attractiveness and preference (bias) for the infected host. The sick time, recovery time, incubation time, and carrier time are modeled with random normal distributions, with average times as specified in Table 1 and standard deviation set to one fourth of the average, to allow some variability in the infection and transmission parameters, which is often the case in real scenarios.

### 2.2 Grid search for *attractiveness* and *bias*

To study the impact of attractiveness to human hosts and bias of mosquitoes for infected individuals, we performed a systematic grid search for attractiveness and bias parameter values in the range from 0 to 1.0, with 0.1 as step size. Low values of attractiveness indicate mosquitoes having little to no preference to move toward human hosts. Similarly, low values of bias correspond to little to no preference for infected individuals.

### 2.3 Sensitivity analysis

Parameters that were taken from the literature were kept constant for all simulation runs. For instance, average sick time, average carrier time, recovery time and average incubation time were set to 5, 21, 30, and 7 days, respectively (Table 1). In order to determine the sensitivity of the model to changes in parameter values, we performed simulations with a range of parameter values for percentage infected at start and mosquito population. The human population was set to 100, as larger values resulted in an overcrowded environment, in which mosquitoes can always find a host, irrespective of bias, and attractiveness.

## 3 Results

**Figure 1:**
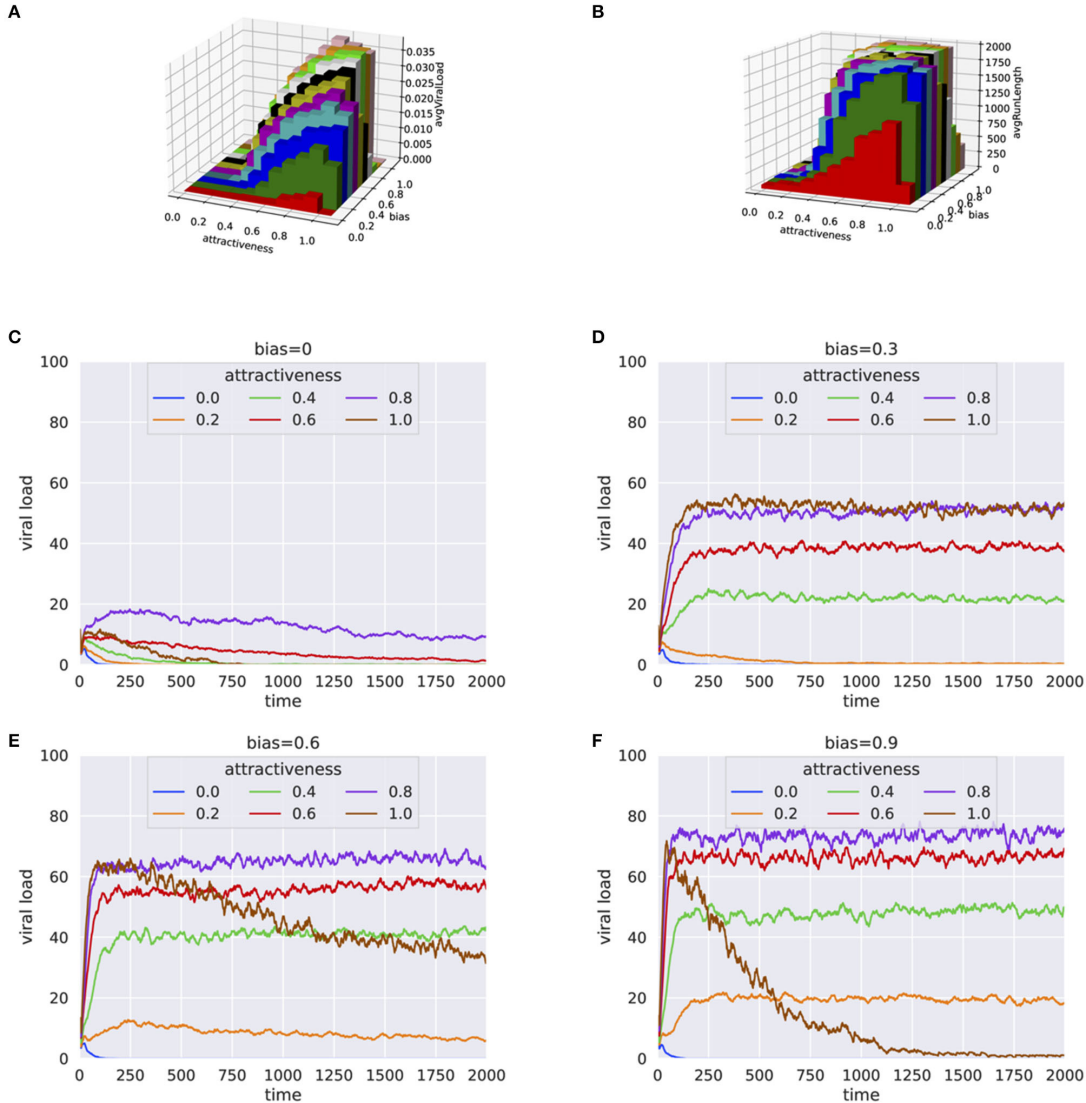
Results from simulation experiments plotting **(A)** Average viral load vs. human attractiveness and bias **(B)** Average simulation time vs. human attractiveness and bias **(C–F)** Average viral load over each step for varying human attractiveness and *bias* = 0.0, 0.3, 0.6, and 0.9, respectively. The height of 3-D bars in **(A, B)** represents the average viral load.

Simulation experiments based on systematic grid search for human attractiveness and bias were carried out. The simulation results included simulation time and viral load at the end of each simulation. For every pair of human attractiveness and bias values, we plotted average viral load and average simulation run length (Figures 1A, B and Supplementary Figures 4–9). Additionally, the trend in viral transmission is shown by plotting viral load over time (see Figures 1C–F).

When there is no preference of the mosquitoes for infected hosts (*bias* = 0), the viral load in the population either died off quickly or remained at a low level (Figures 1A, B), even when human attractiveness was set to high. This trend was consistent even when the percentage of infected individual was high (e.g., 30%) at the beginning of the simulation (Supplementary Figures 4, 5). When the population of mosquitoes was doubled to 100, similar trends were observed with little to no bias for infected hosts (see Supplementary Figures 6, 7). However, even with small bias values for infected hosts (*bias* = 0.3), the infection persisted for a much longer period (Figure 1D). These results suggests that the preferential bias for infected hosts has a substantial effect on the ability of the virus to propagate in the population.

The results were consistent for a wide range of parameters for percentage infected at start and number of mosquitoes, as shown by the high correlation between runs (Supplementary Figures 2, 3). In a crowded environment (i.e., with a large number of mosquitoes and humans), we observed persistent infections even with little or no bias for infected hosts. This can be explained by the high probability that mosquitoes will find a host (Supplementary Figures 8, 9).

Generally, an increase in bias and attractiveness resulted in higher viral propagation in the population (Figures 1D, E). However, for extreme values of attractiveness to human and bias (when *attractiveness* = 1 and *bias* = 0.9) toward infected individuals, we observed a sudden decrease of viral load (Figure 1F). One explanation for this behavior is that the mosquitoes end up biting the same infected individuals again and again, with a decreased probability for the infection to spread to more individuals.

## 4 Discussion

Here, we presented an agent-based model that simulates the propagation of a vector-borne viral infection in a population, systematically studying the impact of vector's attractiveness to human and its bias for infected hosts. Our results indicate that without bias for infected hosts, vector-borne diseases can die off relatively quickly. Conversely, infections tend to persist for longer periods of time and viral propagation increases substantially even with a moderate bias for infected individuals. However, very high values of bias and attractiveness lead to reduced viral propagation, as a result of the vectors biting the same infected individuals multiple times.

Our agent-based model does not aim to realistically simulate all the complexities underlying the spread of vector-borne diseases. Rather, it aims to provide an interpretable evolutionary explanation for the preferential attraction of mosquitoes for infected hosts, described in Zhang et al. (2). Further, the results of our simulations clearly suggest that intervention strategies that can decrease or eliminate the vector bias for infected hosts might be very effective in reducing the spread of vector-borne diseases.

## 5 Model availability

The source code for our NetLogo model is available on GitHub at the following URL: https://github.com/ishworthapa/biasInfectionModel. The model can be run in the NetLogo modeling environment, freely available at: https://ccl.northwestern.edu/netlogo/. A snapshot of our NetLogo model interface is shown in Supplementary Figure 1.

## Data availability statement

The original contributions presented in the study are included in the article/Supplementary material, further inquiries can be directed to the corresponding author.

## Author contributions

IT: Conceptualization, Investigation, Methodology, Software, Visualization, Writing—original draft, Writing—review & editing. DG: Conceptualization, Investigation, Methodology, Software, Supervision, Visualization, Writing—original draft, Writing—review & editing.
