# Supplementary material for: Modeling preferential attraction to infected hosts in vector-borne diseases
Source: Front Public Health. 2023 Nov 22;11:1276029. doi: 10.3389/fpubh.2023.1276029 (PMC10710135; doi:10.3389/fpubh.2023.1276029)
Supplement: Supplementary file 1 [file Data_Sheet_1.pdf]

Supplementary materials

Modeling Preferential Attraction to Infected Hosts in Vector-Borne Diseases

Ishwor Thapa and Dario Ghersi
School of Interdisciplinary Informatics,
University of Nebraska at Omaha, Omaha, NE USA

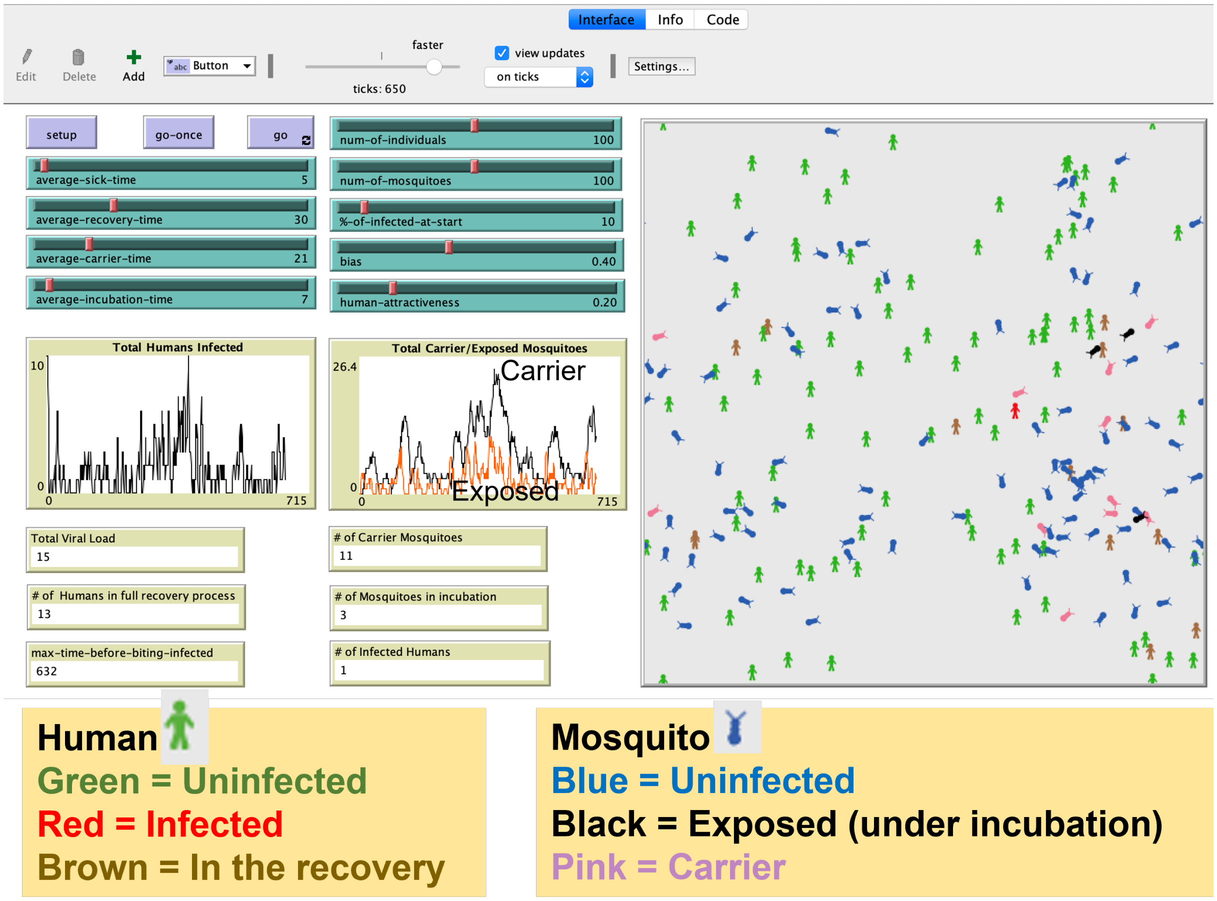

Figure 1: NetLogo Interface with the agent at different states. The color codes for human and mosquito agents based on different states are provided below the screenshot.

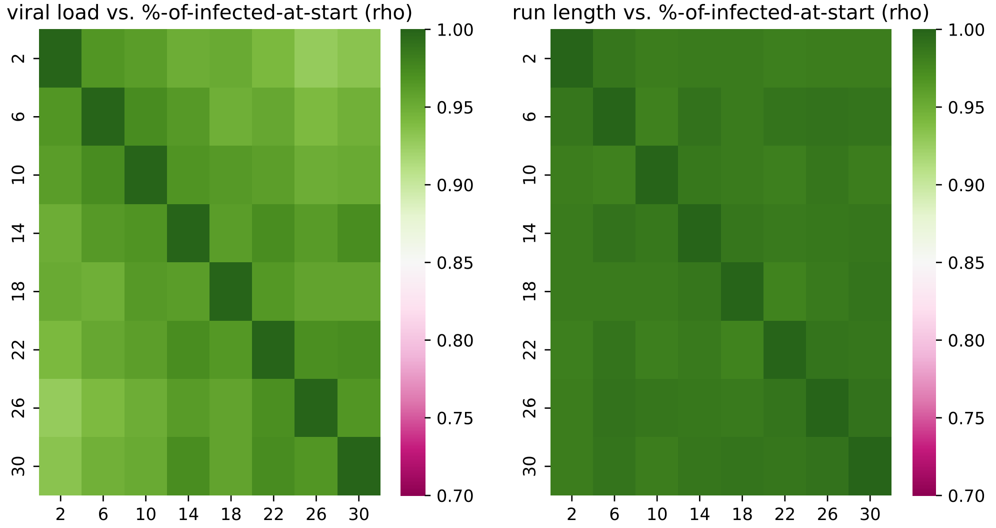

Figure 2: Spearman's Rho value for the viral load (left) and the run length (right) obtained using different values for percentage of infected at start. The $\% - of - infected - at - start$ value ranges from 2 to 30 with an increment of 4. The $num - of - individuals$ and $num - of - mosquitoes$ are set to 100 each

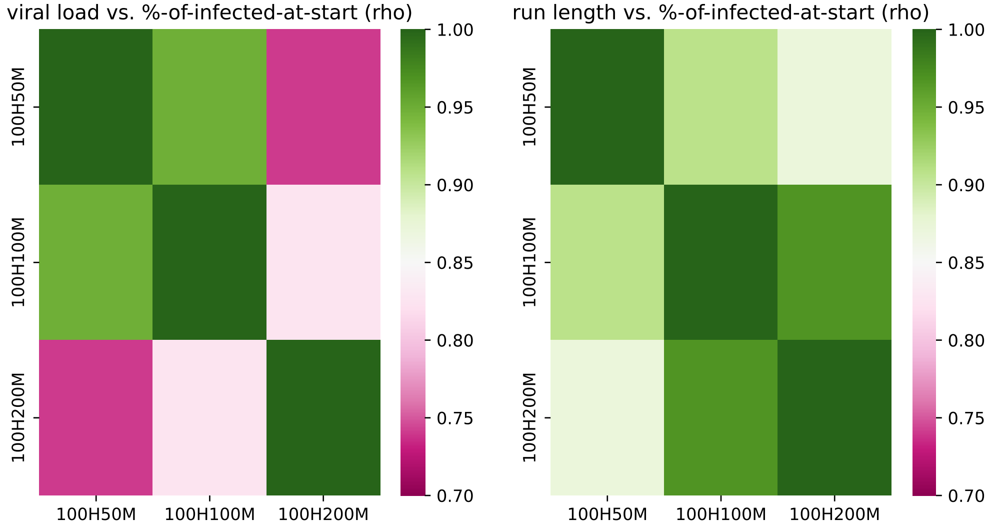

Figure 3: Spearman's Rho value for the viral load (left) and the run length (right) obtained using different numbers of mosquitoes. The $num-of-individuals$ is set to 100 and $num-of-mosquitoes$ is set to 50, 100 and 200 in the simulations labeled as 100H50M, 100H100M and 100H200M, respectively. The $\%-of-infected-at-start$ value is fixed at 10%.

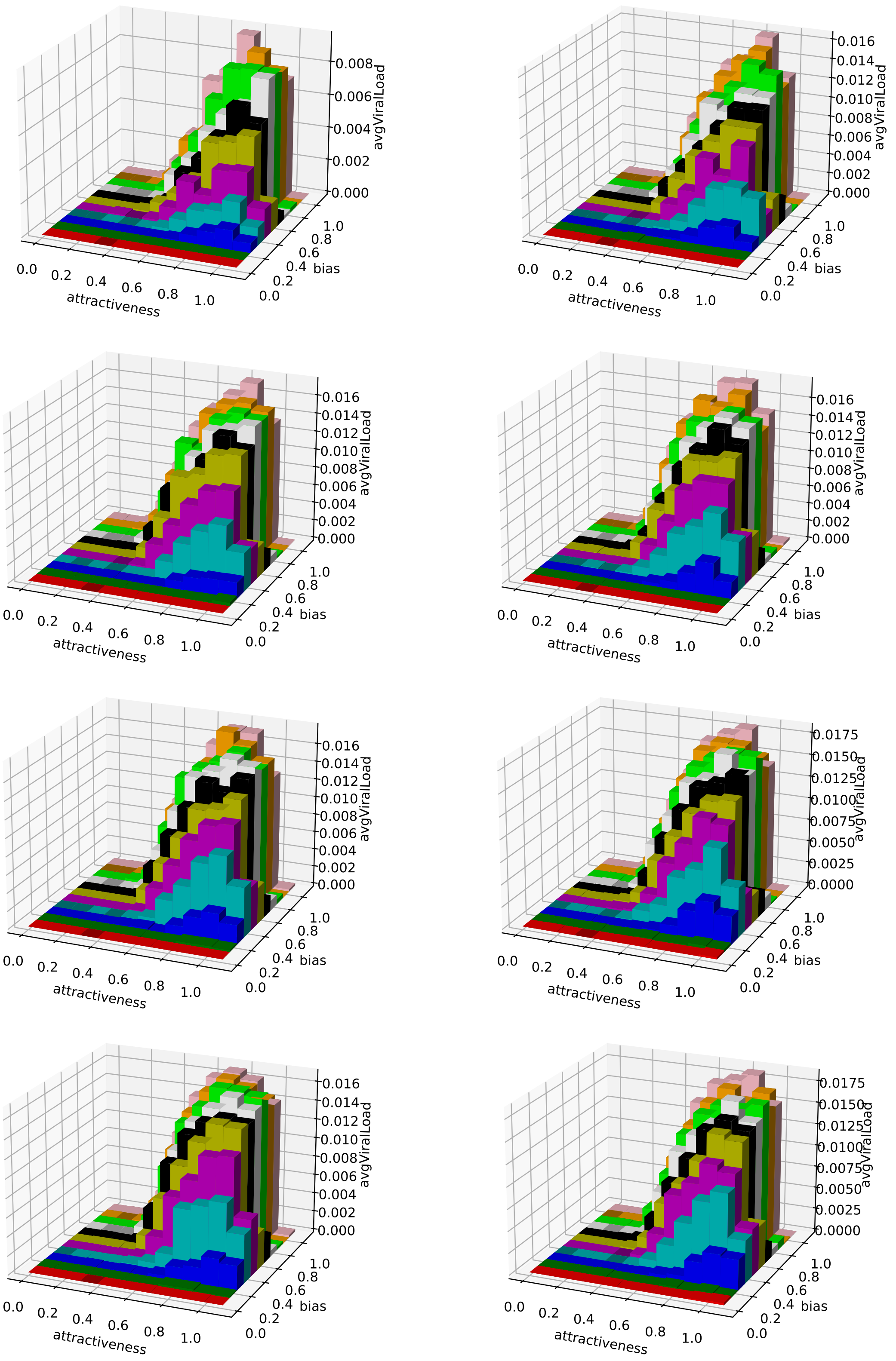

Figure 4: Average viral load for multiple runs where the $\% - of - infected - at - start$ value ranges from 2 to 30 with an increment of 4. For an instance, the values of $\% - of - infected - at - start$ for the top row are 2 (left) and 6 (right). The $num - of - individuals$ is 100 and $num - of - mosquitoes$ is 50.

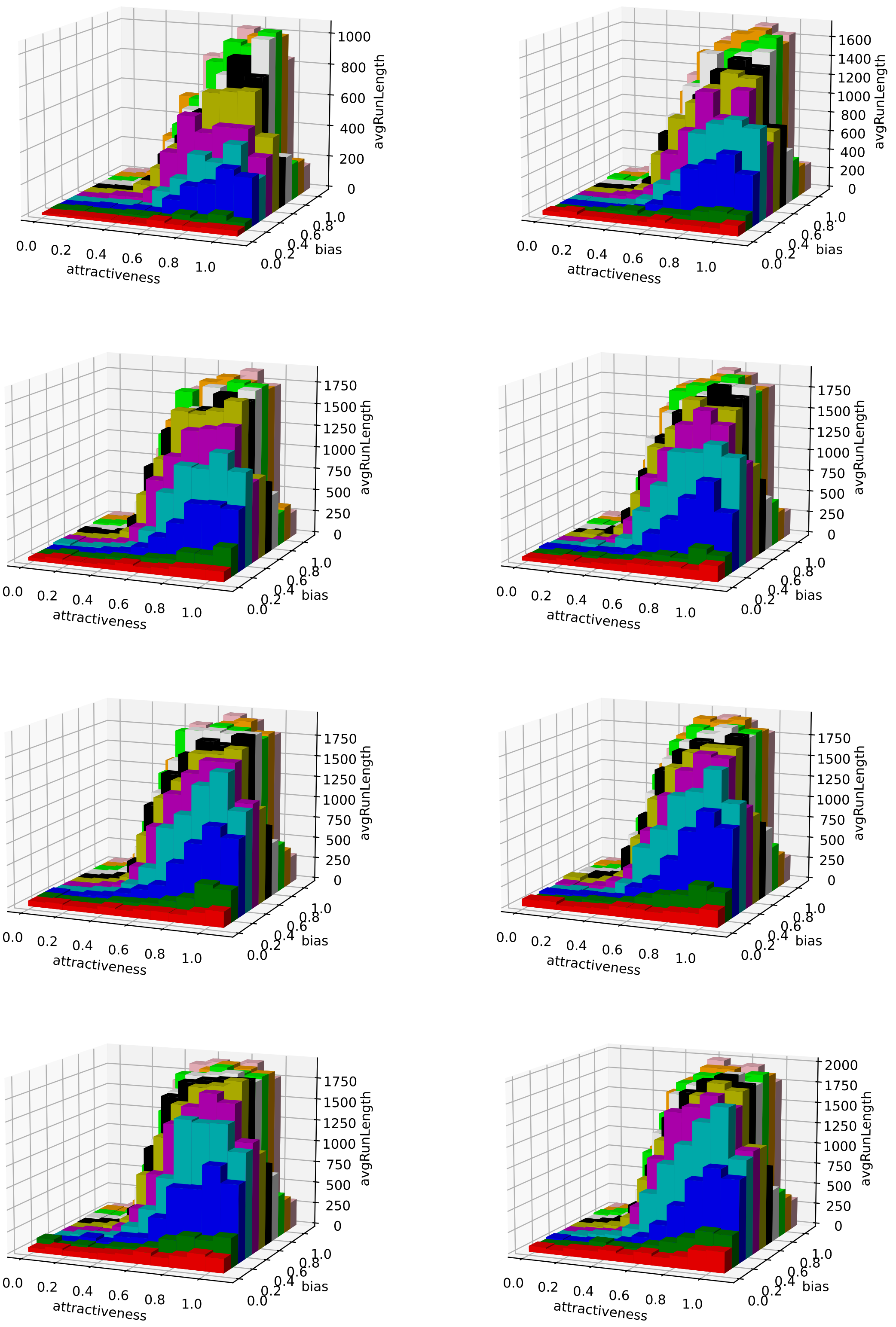

Figure 5: Average run length for multiple runs where the $\% - of - infected - at - start$ value ranges from 2 to 30 with an increment of 4. For an instance, the values of $\% - of - infected - at - start$ for the top row are 2 (left) and 6 (right). The $num - of - individuals$ is 100 and $num - of - mosquitoes$ is 50.

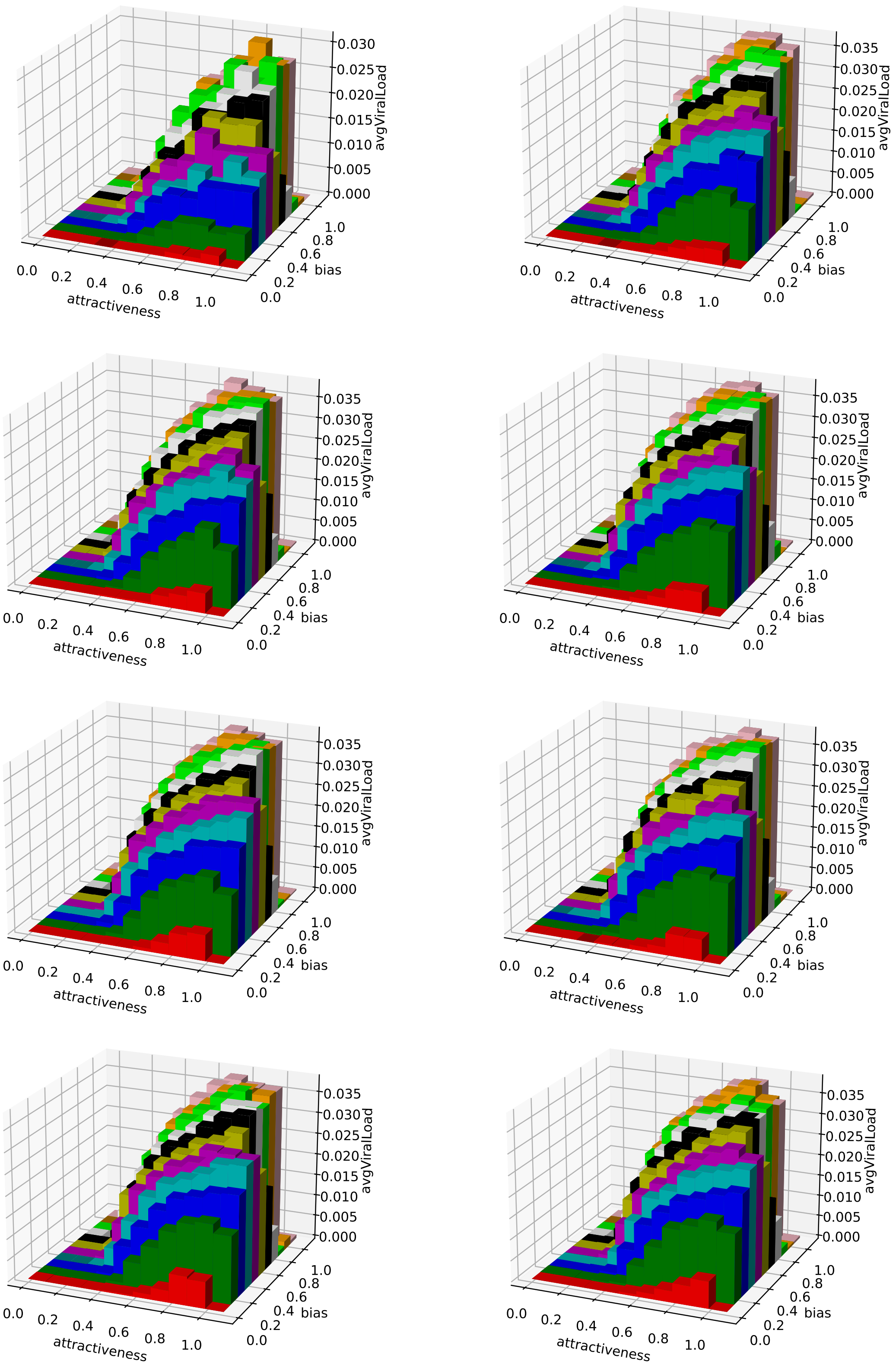

Figure 6: Average viral load for multiple runs where the $\% - of - infected - at - start$ value ranges from 2 to 30 with an increment of 4. For an instance, the values of $\% - of - infected - at - start$ for the top row are 2 (left) and 6 (right). The $num - of - individuals$ and $num - of - mosquitoes$ are set to 100 each.

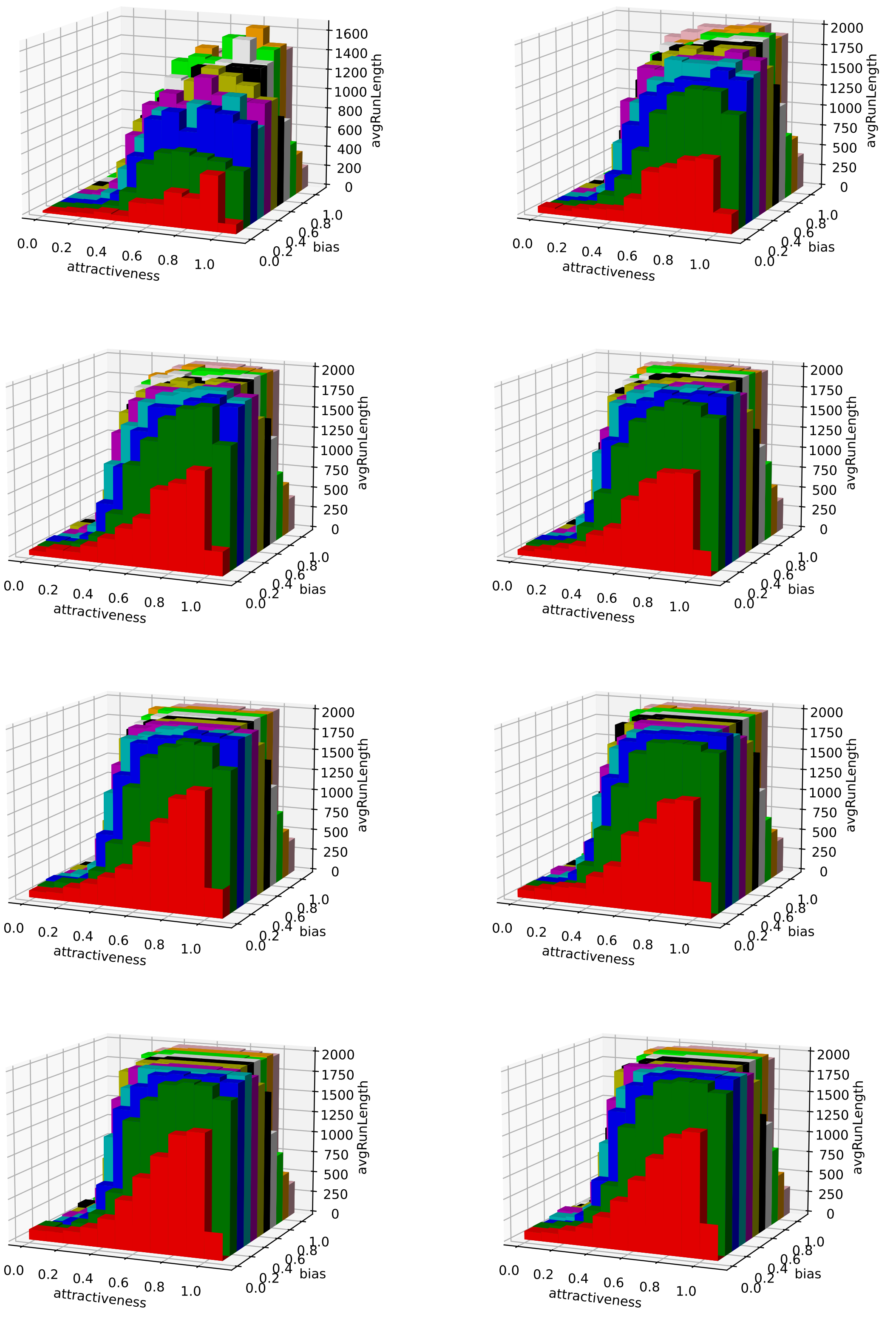

Figure 7: Average run length for multiple runs where the $\% - of - infected - at - start$ value ranges from 2 to 30 with an increment of 4. For an instance, the values of $\% - of - infected - at - start$ for the top row are 2 (left) and 6 (right). The $num - of - individuals$ and $num - of - mosquitoes$ are set to 100 each.

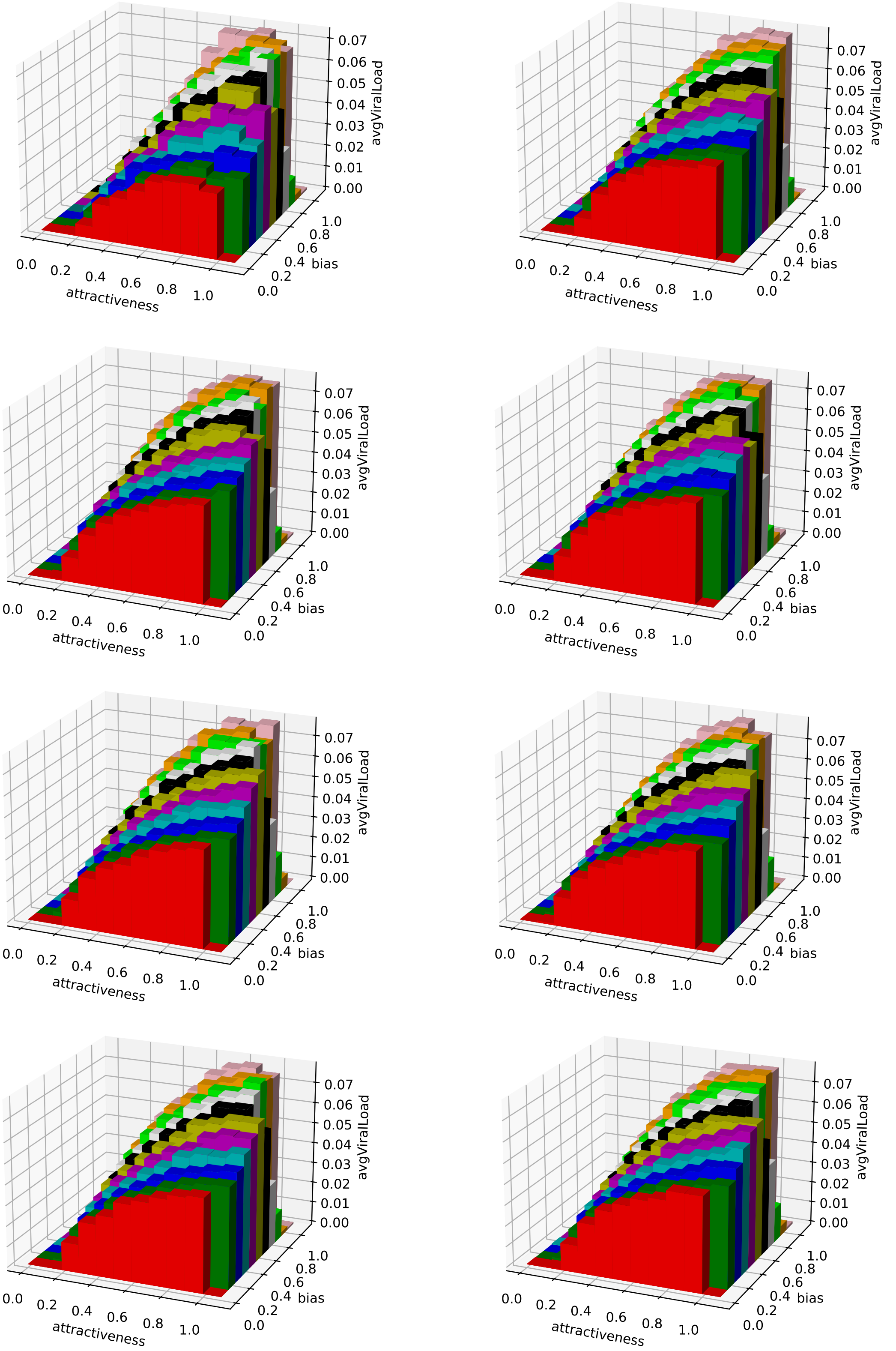

Figure 8: Average viral load for multiple runs where the $\% - of - infected - at - start$ value ranges from 2 to 30 with an increment of 4. For an instance, the values of $\% - of - infected - at - start$ for the top row are 2 (left) and 6 (right). The $num - of - individuals$ is 100 and $num - of - mosquitoes$ is 200.

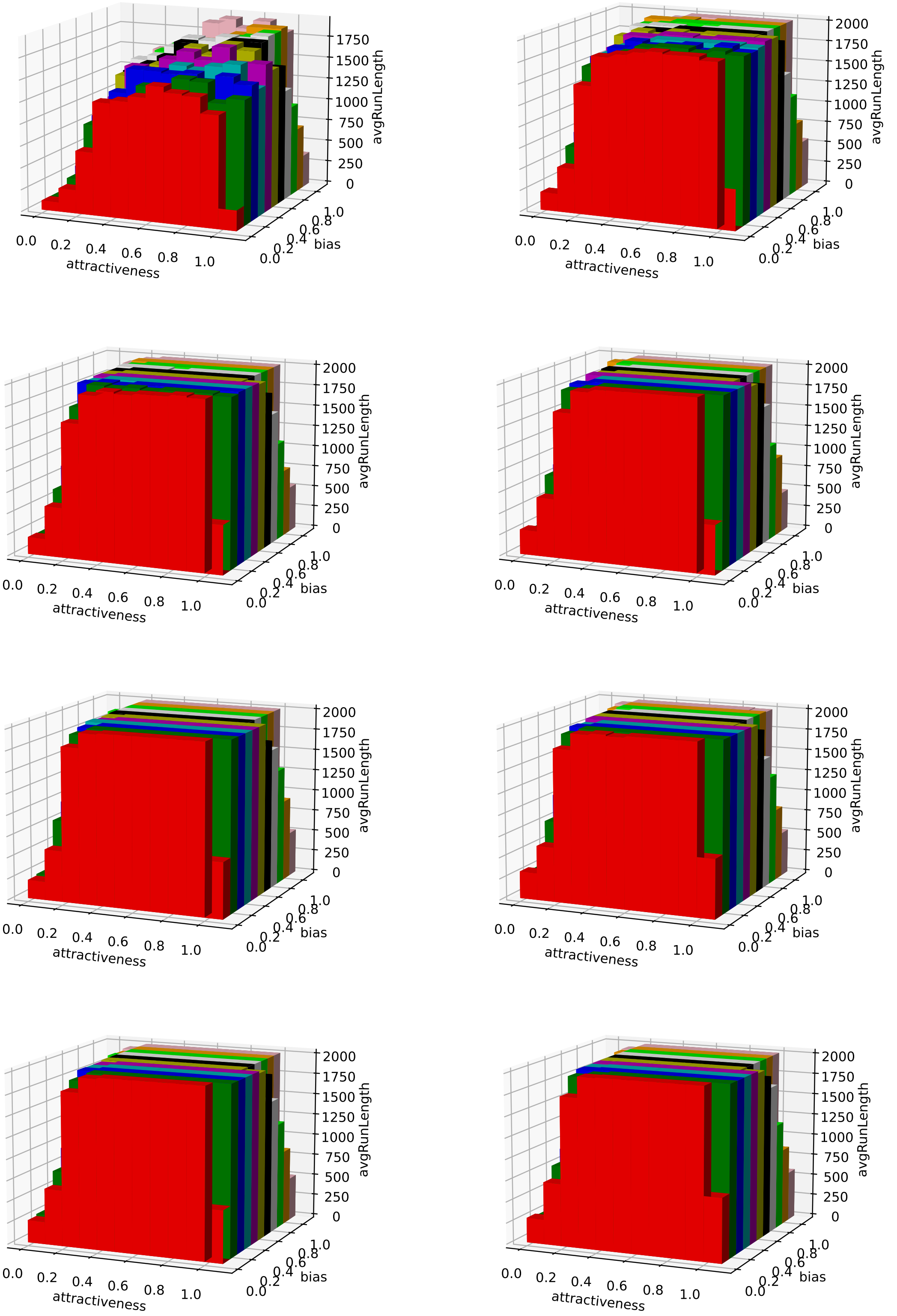

Figure 9: Average run length for multiple runs where the $\% - of - infected - at - start$ value ranges from 2 to 30 with an increment of 4. For an instance, the values of $\% - of - infected - at - start$ for the top row are 2 (left) and 6 (right). The $num - of - individuals$ is 100 and $num - of - mosquitoes$ is 200.
